# Effects of reliability indicators on usage, acceptance and preference of predictive process management decision support systems

**DOI:** 10.1007/s41233-022-00053-0

**Published:** 2022-09-05

**Authors:** Peter Fröhlich, Alexander G. Mirnig, Damiano Falcioni, Johann Schrammel, Lisa Diamond, Isabel Fischer, Manfred Tscheligi

**Affiliations:** 1 Center for Technology Experience, AIT Austrian Institute of Technology, Giefinggasse 2, 1210 Vienna, Austria; 2 Center for Human-Computer Interaction, University of Salzburg, Jakob-Haringer-Straße 8, 5020 Salzburg, Austria; 3 BOC GmbH, Operngasse 20b, 1040 Vienna, Austria; 4 Warwick Business School, University of Warwick, Scarman Rd., Coventry, CV4 7AL UK

**Keywords:** Decision support systems, Trust calibration, Reliability indication, User interfaces, Predictive process management

## Abstract

Despite the growing availability of data, simulation technologies, and predictive analytics, it is not yet clear whether and under which conditions users will trust Decision Support Systems (DSS). DSS are designed to support users in making more informed decisions in specialized tasks through more accurate predictions and recommendations. This mixed-methods user study contributes to the research on trust calibration by analyzing the potential effects of integrated reliability indication in DSS user interfaces for process management in first-time usage situations characterized by uncertainty. Ten experts specialized in digital tools for construction were asked to test and assess two versions of a DSS in a renovation project scenario. We found that while users stated that they need full access to all information to make their own decisions, reliability indication in DSS tends to make users more willing to make preliminary decisions, with users adapting their confidence and reliance to the indicated reliability. Reliability indication in DSS also increases subjective usefulness and system reliability. Based on these findings, it is recommended that for the design of reliability indication practitioners consider displaying a combination of reliability information at several granularity levels in DSS user interfaces, including visualizations, such as a traffic light system, and to also provide explanations for the reliability information. Further research directions towards achieving trustworthy decision support in complex environments are proposed.

## Introduction

Decision support systems (DSS) aim to provide users with tools to enhance their decision making process for semi-structured or unstructured problems [7, 86, 97]. Such systems provide reasoning capabilities based on data and communicate these through a user interface. DSS (also called “decision aids”) have in the last 40 years been used in disciplines such as medicine, manufacturing (e.g., production scheduling and process optimization), environmental management, GIS planning and farming management [49, 61]. With the growing capacities of big data and advanced data processing approaches, such systems have increasingly reached beyond pure descriptive analytics (“what did happen?”) and offer predictions (“what will happen”) and even prescriptive advice (“what should I do?) [58].

DSS are being introduced in an increasing variety of application sectors and the complexity of supported tasks is rising. Process management in the construction industry represents one of the most challenging DSS application areas, as the involved tasks are characterized by high complexity [9] and thus would require sophisticated technological aids to support decisions [1, 45]. This process of digitization is fuelled by building information modelling (BIM), a mostly descriptive form of DSS that enables the fine-grained and dynamic digital representation of physical and functional characteristics of a facility [42]. These forms of decision support systems are also increasingly introduced in the process management of construction projects. Using decision aids by BIM has been suggested to be beneficial for reducing critical mistakes and omissions, and to improve communication between stakeholders involved in construction projects [89].

Despite the increasing capacities of DSS, the level of adoption by users and relevant stakeholders in everyday work practices has found to be still limited [3, 18, 34, 41, 52, 59]. Also in the construction industry, concerns have so far hindered wide uptake of digitized decision support [36], namely the necessity for all stakeholders to use consistent tools and formats, the learning curve to get accustomed to novel technologies, as well as the large data size required for BIM [83].

One of the most decisive factors for the acceptance and uptake of predictive DSS in general is the trust in the decisions and underlying knowledge base [97]. Trust is defined as a relation between two or more agents, where one or more agents (trustors) depend on the performance of another agent’s (trustee) goals in a situational context characterized by uncertainty and vulnerability [56]. Overtrust in a system can happen when the perceived capability is higher than the actual capability, whereas undertrust implies that the perceived capabilities are lower than the actual capabilities. Users can underestimate the likelihood that a system will make serious mistakes at all, but they can as well underestimate the consequences if a system fails. Trust in a system is calibrated well when neither over- nor undertrust occur. In construction and renovation projects, practitioners traditionally have limited trust in the underlying data [9, 64]. A major factor for the limited trust is the uncertainty and complexity of construction projects, which can be defined as “the chance of the occurrence of some event where probability distribution is genuinely not known” [111]. Factors for uncertainty in construction projects may be ascribed to a lack of uniformity due to constantly changing resources, the effects of climate and weather, and the lack of experienced workforce.

## Intended contribution and research questions

The intended contribution of this paper is to demonstrate the effects of reliability indication on prediction strategies, decision confidence, trust, and acceptance in complex process management under unfamiliar and uncertain conditions. Furthermore, insights shall be derived with regard to the information requirements for reliability indication, both regarding information type and presentation granularity in the user interface. The mixed methods approach pursued for this purpose contributes with the systematic investigation of the effects of reliability indication in concrete usage situations (through an experimental setup capturing both quantitative and qualitative data), the measurement of technology acceptance and trust (using an inventory adapted for the specific purpose of the study) and the comparative evaluation of novel integrated concepts for reliability indication (through experience prototyping and reliability modeling). The paper aims to provide an empirical research contribution (cf. [109]) to the field of Human-Computer Interaction (HCI). The research is contextualized in process management for building renovation, and it seeks to generate conclusions for similar tasks and environments characterized by complexity and uncertainty. The specific research questions (RQ) are as follows:

- **RQ1:** Does the indication of reliability in Predictive decision support systems have effects on users’
  - prediction strategies, and
  - confidence in these predictions

in the context of renovation process management (and if yes, how can the effects be described)?

- **RQ2:** Does the indication of reliability have effects on trust and acceptance of Predictive DSS in the context of renovation process management (and if yes, how can the effects be described)?
- **RQ3:** Which and how much information should reliability indicators provide when integrated in predictive decision support systems for renovation process management, such that their users are optimally supported in calibrating their trust?

The remaining paper is structured in six parts. First, the literature about trust and acceptance theories, approaches and empirical evaluations is presented. Then, in Sect. "Methodology" the overall study design, the participant characteristics, and the procedure of the experimental study is described. In Sect. "Results", the findings from the different phases of the study are presented, followed by their discussion in relation to previous research in Sect. "Discussion". The concluding Sect. "Conclusions" proposes implications of the study in various respects, critically reviews the limitations of the chosen approach, and suggests further research activities towards effective and satisfactory trust calibration in predictive DSS.

## Background and related work

Predictive DSS involve different forms of intelligent processing and automated reasoning [85]. While their current level of automation varies, the role of their operators is increasingly transcending from active usage towards more “passive” supervisory control (compare characteristics of automation experience in [33]. Building on Janssen et al. (2020), [33] classify decision support systems as an automated system, similar to the classical notion of expert systems, involving knowledge acquisition components, and typically operating in well-defined application areas. Acceptance and trust are key requirements when considering human-automation interaction and we therefore explain in the following sections, important aspects of technology acceptance as well as trust, reliance and trust calibration.

**Fig. 1:**
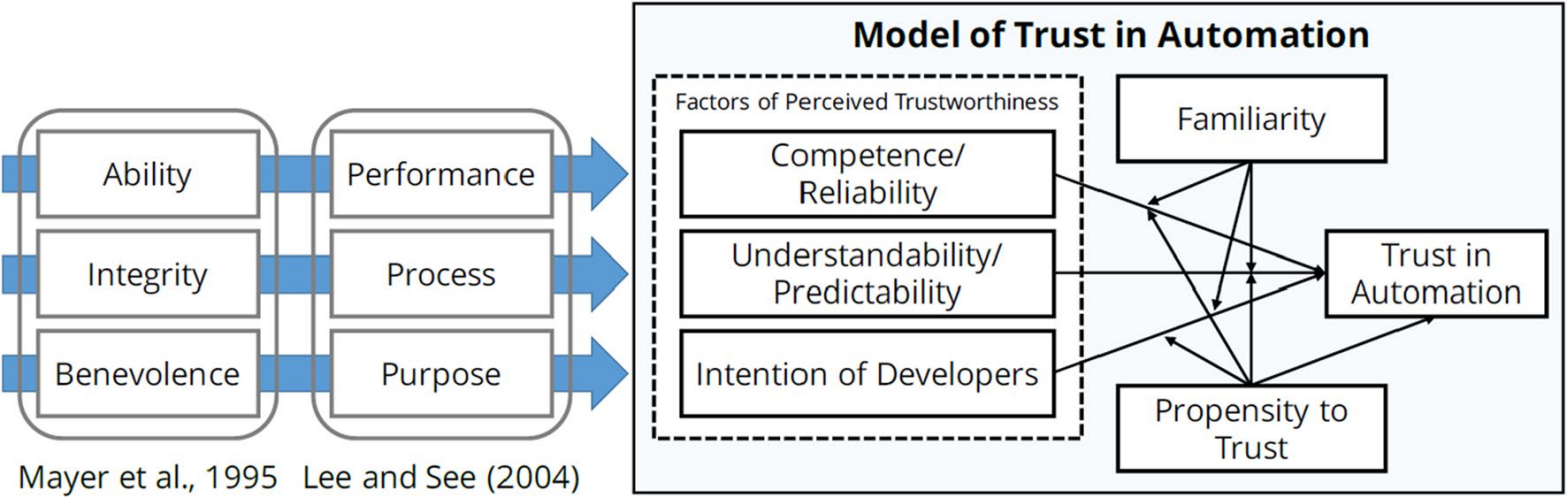
Model of trust in automation [51], which is based on the postulated dimensions by Lee and See [56, 65]

### Technology acceptance

It has widely been acknowledged that user acceptance is a major determinant for adoption of information technology (IT) in general, and different models have been proposed to explain the determinants for acceptance and intention to use [98]. User acceptance can be defined as ”the demonstrable willingness within a user group to employ information technology for the tasks it is designed to support” [24]. Many theories for describing and explaining innovation diffusion have been proposed (e.g., [88] with an organizational scope, which does not account for the individual perspective of information systems [13, 97]. Due to its orientation towards individual experience, the Technology Acceptance Model (TAM), originally introduced by Davis [22, 23], has been regarded as the framework of choice when investigating acceptance in various application fields [2].

Other models have meanwhile extended the TAM model, one prominent example being the Unified Theory of Acceptance and Use of Technology (UTAUT; [101]). It adds performance and effort expectancy, social influence, facilitating conditions, and sociodemographic factors to the two factors perceived usefulness and perceived ease of use of the TAM model. There are other general models as well as a substantial number of technology- and/or sector-specific specialized models (see an overview in [2, 102]).

For the more specific system type of DSS, tailored technology acceptance models have been proposed, however not yet in the area of construction. Shibl et al. [97] present a technology acceptance model for DSS that is based on the above mentioned UTAUT model [101]. Besides specific usefulness and ease of use factors from the clinical sector, the authors stress the trust in the knowledge base of a DSS as an important determinant for the behavioral intention to use it. While the original TAM and its successor models do not feature trust as a first-level component, the investigation of intelligent, automated and safety critical systems stresses trust more strongly [57]. This is reflected by some of the adapted TAM versions that deal with automated and intelligent systems, also highlighting the role of trust as an important antecedent for user intention [32, 103].

A recent meta analysis demonstrates the wealth of factors that have found to be associated with technology acceptance, comprising 21 variables with 132 intercorrelations [25]. However, also limitations of technology acceptance modeling have been highlighted [16], most importantly the reliance on self-reported use data (instead of observing actual usage), the pure focus on a user’s intention (neglecting contextual factors) and the limited practical value of abstract factors and their correlations. It therefore appears important to apply mixed method study designs, by combining technology acceptance with observation-based methods and qualitative data gathering.

### Trust and reliance

There have been many approaches towards the definition and measurement of trust [46, 63, 82, 112]. Trust is seen as a construct that is relevant both for relationships among humans and between humans and machines. A frequently used definition of trust stems from the model of organizational trust by Mayer, Davis and Schoorman [65], p. 712): “the willingness of a party to be vulnerable to the actions of another party based on the expectation that the other will perform a particular action important to the trustor, irrespective of the ability to monitor or control that other party.”

As the model by Mayer et al. [65] is related to interpersonal trust, Lee and See [56] adapted it to better account for human-automation interaction. Figure 1, based on Körber [51], shows the alignment between their respective models. The three dimensions of Lee and See [56]—*performance*, *process*, and *purpose*—correspond to the trustworthiness *ability*, *integrity*, and *benevolence* from Mayer et al. [65]. Körber [51] takes this further and operationalizes these constructs and derives a questionnaire for trust in automation. As shown in the figure, the sub-scales of this questionnaire comprise items on competence and reliability (corresponding to the performance dimension by Lee and See [56], understandability and predictability for the process dimension, and intention of developers for purpose.

**Table 1:** Requirements for communicating data reliability in Predictive DSS for construction and renovation management, as derived by Mirnig et al. [70]

	Information requirement	Description
1	Input type	Who or what provided an input, estimation or prediction? (method, the used system, the user role, level of expertise)
2	Timeliness of input	Time period of data input: longer delays make data reliability less likely
3	Penalty applying	In case of existence of such a penalty, a higher reliability is ascribed to it by project managers
4	Appropriateness of abstraction level	Follow the degree of abstraction: highlight upfront the level of detail of the model
5	Properties and restrictions	Highlight the nature, properties and restrictions/uncertainties of the underlying model
6	Customization: Filtering	It should be possible to filter system output (both with regard to data protection and to usability)
7	Customization: specification of input provider	It should be possible to define who provided an input and the related chosen approach
8	Explainable AI	In the case that AI is involved in the data processing, provide an indication on which underlying data and models are used
9	Previous reliability checks	The system should inform on whether the considered building model or respective estimations have undergone previous reliability checks

### Strategies for trust calibration

Designing effective strategies for trust calibration requires knowing what information is needed to support trust calibration [26]. [70] identified nine factors that are most indicative for enabling the inspection of data reliability in concrete usage situations in the BIM-context, based on qualitative interviews with experts (see Table 1. Following a prioritization based on effectiveness and feasibility for user interface integration, [70] present design patterns for the three highest priority requirements: input type, timeliness of input and contractual penalty.

Different approaches for fostering trust calibration in user interfaces have been presented. Taking [95] as a starting point, these can be categorized in the communication of (1) uncertainty, (2) reliability, (3) awareness and intent, (4) alternatives, and (5) explanations. A direct way of indicating the trustworthiness of a system’s operation or recommendation is to provide a reliability display on the user interface. Often, this is realized by a percentage value or an icon (e.g., a “fill level indicator”). Such as cue often needs to be tailored to the application domain. In map visualizations, the reliability regarding location is typically shown as a circle surrounding the current position [113]. In the context of autonomous driving, the reliability of a system can be shown as an indicator bar beside the main instruments on the car’s dashboard [19, 20, 43, 44, 53]. A different type of reliability display can be found in military battlefield visualization: Pie charts or color density are frequently being used to visualize reliability of the friend/enemy detection in combat situations [76, 107].

**Fig. 2:**
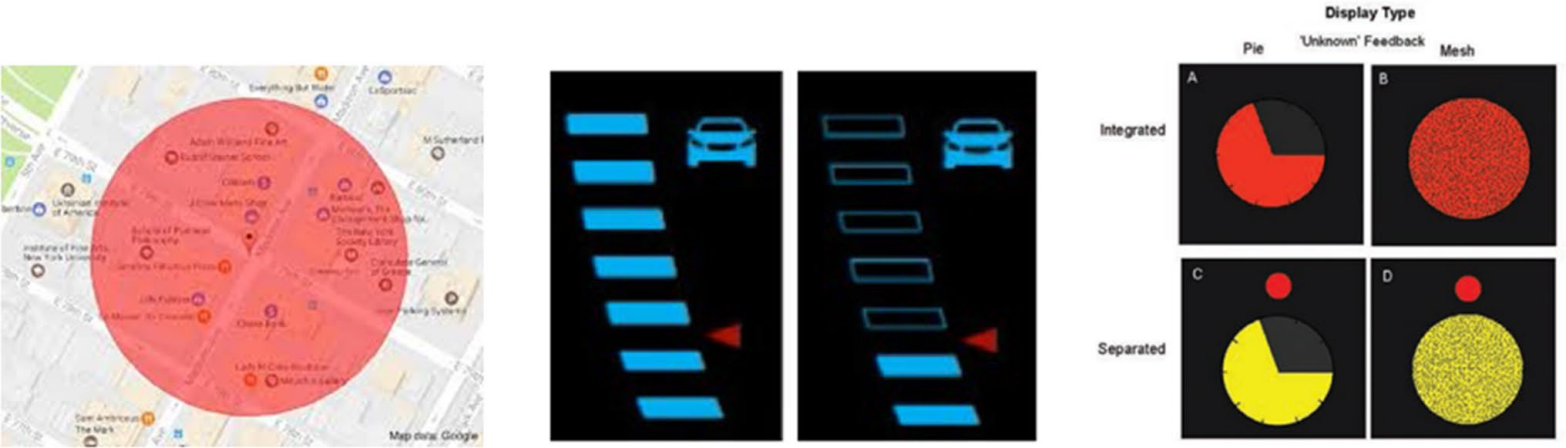
Examples for Communicating Reliability. *Left*: Location positioning reliability [113], *center*: driver assistance [53], *right*: reliability visualizations of friend/enemy detection in combat situations [76]

**Fig. 3:**
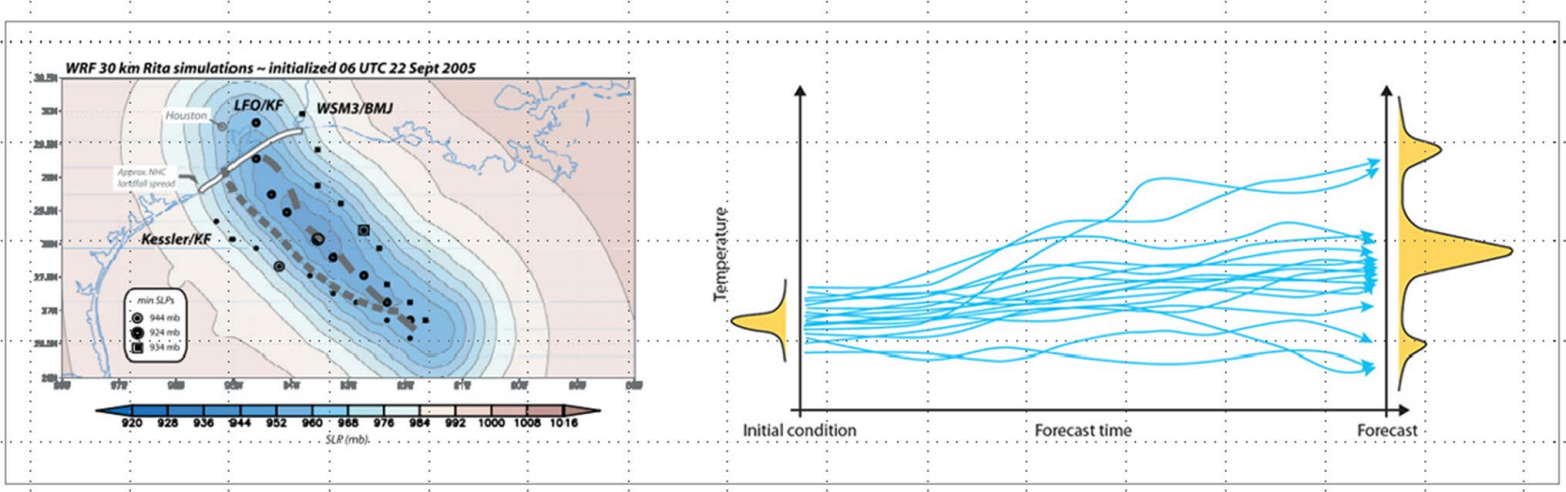
Examples for communicating uncertainty: *Left*: Different ways of showing probability distribution for uncertainty of bus departure times [48]; *right*: showing uncertainty in ensemble weather predictions [39]

Another important approach towards supporting trust calibration is to correctly communicate the underlying level of uncertainty. Communicating uncertainty has been addressed in research in general [11, 38], and is a common problem across many domains, such as e.g. weather forecasts (e.g., [47, 73] or data visualizations (e.g., [21], with learnings from these domains being used to inform trust calibration. Figs. 2 and 3 show example visualizations for communicating uncertainty in these two domains. Typically, probabilities are being used when communicating uncertainty. One challenge when applying this approach is that even well-educated adults have difficulties to solve simple probability questions [60]. To avoid these problems, qualitative information in labels (e.g. “low uncertainty”) have been proposed, but they also can be misleading [106]. In addition, whether uncertainty is formulated negatively or positively also has a considerable influence on the following decision-making process [67]. While no simple one-size-fits-all recommendation can be deducted from previous work, and communication needs to be tailored towards the case at hand, there seems to be consensus that communicating uncertainty leads to better decisions.

Communicating awareness and intent has mainly been researched in two application domains so far: autonomous driving and human robot interaction. While some studies call for explicit interfaces to communicate awareness and intent [12, 17, 62, 71] other studies suggest that for routine situations implicit communication (via movement of the vehicle or robot) might be sufficient [90].

In DSS, which are increasingly driven by artificial intelligence, transparency cues [108] as well as explanations of the underlying data and algorithms have been investigated as a means to establish trust by users [94]. In a systematic review of 217 studies, [79] found that the explanations in most of the studies are strongly related to the underlying reasoning process and thus have a strong engineering root. However, also interdisciplinary approaches have been presented that incorporate cognitive psychology and philosophy in order to reframe and reflect the motivations and reasons why explanations are to be used in intelligent systems [54]. Recently, there have been approaches to design for explainability of advanced machine learning to non-expert users [15, 27, 75]. So far, the learnings have not been transferred to specific application areas, such as construction.

Another important element for DSS usage is to communicate possible action alternatives with high probability to the human user. This allows the user to develop expectations regarding possible action outcomes, and to prepare for interventions and taking over control in case the anticipated actions are problematic. In prior work similar problems have been addressed, for example, from the perspective of comparative data visualizations [69] and also DSS [74, 105]. The results from the data visualization research domain appear especially well suited to assist in designing information systems for communicating action alternatives for trust calibration. van der Waa et al. [104] present a framework for making confidence explanations in DSS interpretable. They postulate that explanations need to (1) be accurate, (2) be able to explain an arbitrary single datapoint to the user, (3) use a transparent algorithm, and (4) provide confidence that are predictable for humans.

### Effectiveness of trust calibration communication

While trust calibration cues in interactive computing systems are not widespread in current research or are reflected in market-ready systems, the body of research provides indicative communication strategies for trust calibration. A considerable number of studies suggest positive effects for reliability indicators, e.g., for a summarizing value showing the likelihood of the success of a system, or a respective system function [35]. Antifakos et al. [4] found that for a context-aware memory aid, with varying uncertainty conditions of context recognition, human performance in a memory task is increased when the uncertainty information is explicitly increased. The analysis of a video-based experiment of mobile device usage by Mayer et al. [5] showed that people rely more on the system when reliability information is shown. McGuirl and Sarter [66] found that the display of updated confidence values made aircraft pilots recalibrate their decisions about in-flight icing encounters and led them to more accurate decisions, as compared to when using an overall reliability value. Okamura and Yamada [81] provide evidence that the adaptive presentation of trust calibration cues when undertrust or distrust is detected by the system may provide a more effective approach than a standard continuous trust calibration cue presentation. Chen et al. [14] demonstrate effects trust calibration cues and confirm the central role of calibrated trust also for the application context of IT security.

Other studies draw a more differentiated picture of the effects of reliability cues. For example, [8] found that providing information about reliability increases awareness about the automated system, but it did not show significant effects on users’ trust. [31] did not find evidence for an improvement of sonar detection tasks through providing confidence cues. Rukzio et al. [91] evaluated an automated form filling form for mobile devices with reliability indicators and found that while users spent slightly more time, they committed more errors, as compared to a standard version without the reliability indicators. [54] show positive effects of explanations when they are combined with confidence indicators. Zhang et al. [114] show that presenting confidence scores can enable calibration of users’ trust in an AI-assisted decision-making model, which can assist human experts in applying their knowledge to improve final decision results. Interestingly, the analysis by Zhang et al. [114] represents only one of few studies that consider the impact on domain experts rather than end-users. For clinical decision support systems, [75] found that explanations did not always effectively support users in calibrating their trust, due to conflicts with usability and required efforts.

xplainability for trust calibration might conflict with usability: trust calibration requires extra efforts from users, such as reading and interacting with the explanation.

Displaying reliability or uncertainty in DSS has so far not yet been systematically regarded throughout application sectors. For the area of digital farming, for example, [40] state displaying uncertainty to be very promising but also that with only two studies in this area [30, 92] the empirical basis for this is still insufficient.

**Fig. 4:**
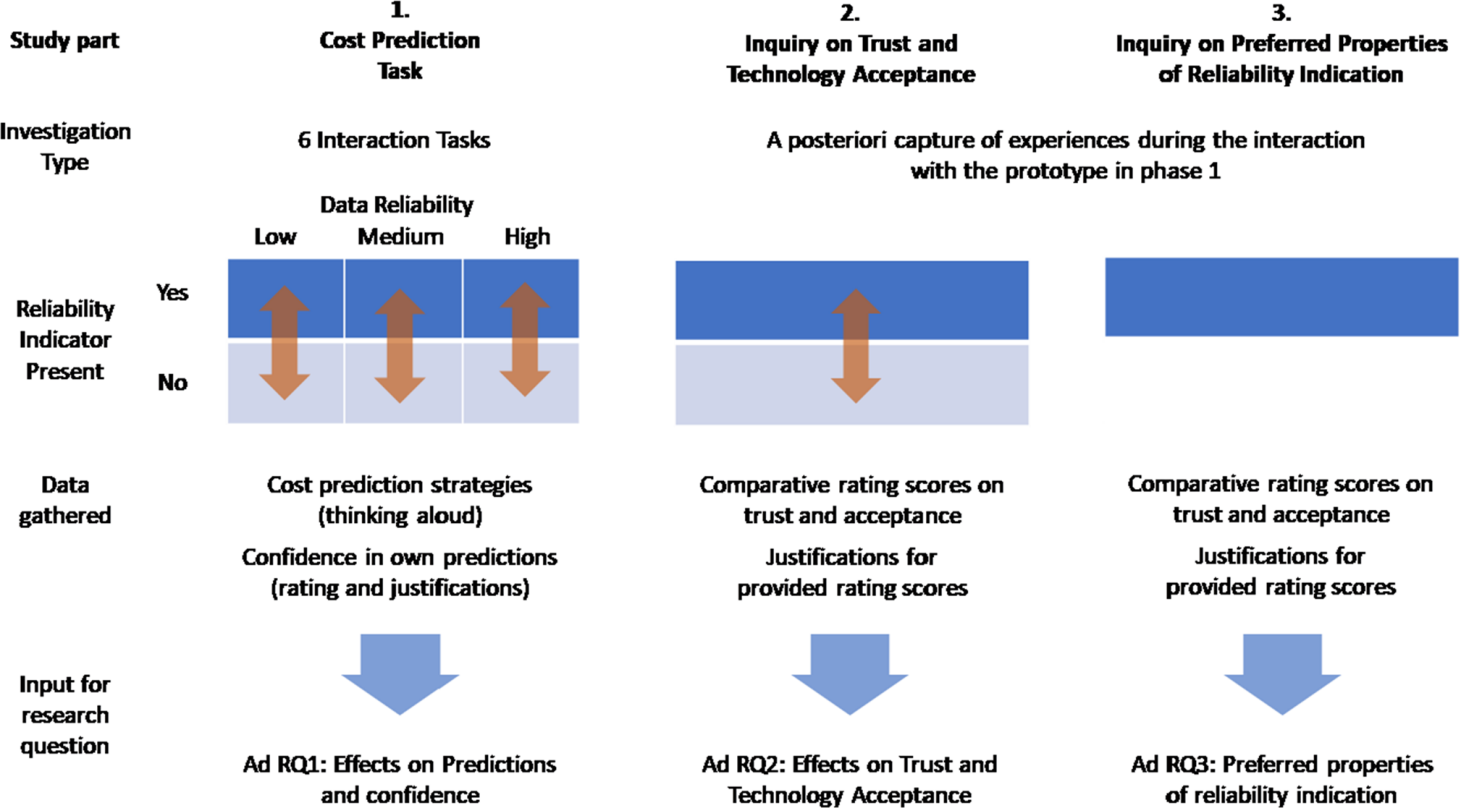
Overview of study parts, the respective investigation type, the involved conditions (presence of reliability indicator, data reliability), the types of gathered data and the expected results for addressing specific research questions

### Summary

In the last 20 years, trust calibration of intelligent and automated systems has received an increasing interest, however so far without systematic application and integration in industry products. As of now, it remains difficult to derive general implications for user interface design, or to tailor these to specific application domains. Given the narrow perspectives taken by previous studies on trust calibration, there is not yet a clear indication on the impact of reliability cues on the user interface of a DSS overall, or on its impact on the usefulness and intention to use in specific application contexts.

From a methodological perspective, most studies so far have not included domain experts as test subjects but have invited end-user representatives via crowdsourcing applications. Thus, currently no valid insights from the perspective of project managers is available, and contextualization of the results to construction projects is so far not possible.

## Methodology

The methodology is grounded in the interdisciplinary research methods compendium of HCI (see an overview in [55] and follows an equal-status sequential multi-phase mixed-methods approach [99] with qualitative methods complementing quantitative results for each of the three research phases: (quan$\rightarrow$qual)$\rightarrow$(quan$\rightarrow$qual)$\rightarrow$(quan$\rightarrow$qual). Within the available “scientific design space of HCI” [87], a method was tailored to investigate the research questions with a balanced goal of internal validity and external validity [50]. Internal validity was accounted for through an experimental design, and external validity was considered by the definition of a concrete and realistic usage scenario, by the participation of expert practitioners from the construction domain (see Sect. "Participant sample", "Technology acceptance"). Using functional web-based interactive prototypes instead of abstract concepts or paper prototypes facilitated richer statements by expert users [93]. In order to capture individual interaction behaviors, preferences and expectations, individual sessions were organized for each participating domain expert (n = 10) instead of a group investigation setting. Due to the COVID-19 pandemic, the user study was conducted online, using a teleconferencing tool that allowed for screen capture and handover of the moderator role to enable participants’ interaction with the web-based prototype. In compliance with European data protection regulations [28] and the ethics requirements of the university of the researchers, participants were informed about the study contents and their rights, and their signed consent forms have been securely stored.

Each of the three main phases of the mixed-methods study was designed to address one of the three research questions (see Fig. 4. In the prediction strategies task that address the effects of reliability indicators on users’ prediction strategies (RQ1) participants performed cost prediction tasks with a dedicated DSS experimental prototype and reported their reasoning and experience using the “thinking aloud method” [77]. In order to understand effects on trust and acceptance (RQ2), an inquiry consisting of a comparative questionnaire and an accompanying interview was conducted to capture the overall reflections from DSS system usage in the prediction strategies-phase (see Sect. "Strategies for trust calibration". The third and final part was an inquiry on the preferred properties of reliability indicators (RQ3).

**Table 2:** Overview of Expert Participant Sample

	Education	Professional role	Experience (Years)	Phases of involvement	BIM experience (Years) current usage of BIM (%)
1	Architecture	BIM Specialist	10	Initiation,planning, education	7 (100)
2	Architecture	BIM Specialist	10	Research,education, standardization	8 (50)
3	Architecture	BIM Specialist	5	Planningphase	15 (50)
4	Construction Engineering	CorporateBIM Strategist	18	Strategy,project monitoring	8 (10)
5	Construction Engineering	Project Manager	2	Construction execution	5 (100)
6	Construction Engineering	Project Manager	27	Construction execution	3 (5)
7	Architecture	BIM Manager	3	BIM-coordination and support of project management	3 (80)
8	Architecture, Construction Engineering	BIM Manager	13	BIM-coordination and support of project management	10 (50)
9	Construction Engineering	Contractand Risk Manager	5	Offer,planning and contract phases	2 (15)
10	Construction Engineering	BIM Manager	15	Offer,planning, contract execution phases	10 (100)

### Participant sample

In order to best account for the target users of predictive DSS in the construction industry, the sample was defined to be experts in digital construction management and building information modeling (BIM). Candidates matching this requirement were identified and contacted through announcements by the national specialist innovation platform for BIM in construction and building management, the own professional network, as well as professional social networks. As Table 2 indicates, the sample comprised an average experience of 11 years working in the construction sector, ranging from long-standing managers who have continuously evaluated and appropriated the domain’s recent digitalization trend (longest experience of 27 years) to BIM experts entering the professional sector after their educational specialization (minimum of 2 years’ experience). Also, the form of involvement with BIM in ongoing projects was varied, ranging from 5% (for the corporate BIM strategist and overall coordinator) to 100% for BIM specialists (average: 50%). The mean age was 38.5 years, with a minimum of 29 years and a maximum of 56 years. The educational background comprised architects and construction engineers. The involvement in construction roles and phases ranged from knowledge transfer, strategy and risk management to project planning, expert accompaniment as BIM-expert to full project management. An equal gender distribution was a dedicated target during the participant recruitment process but the response rate of female participants was low. Nevertheless, the resulting distribution of nine male and one female participant is higher than the current gender distribution in the European construction sector [78, 96].

### Cost prediction tasks

**Fig. 5:**
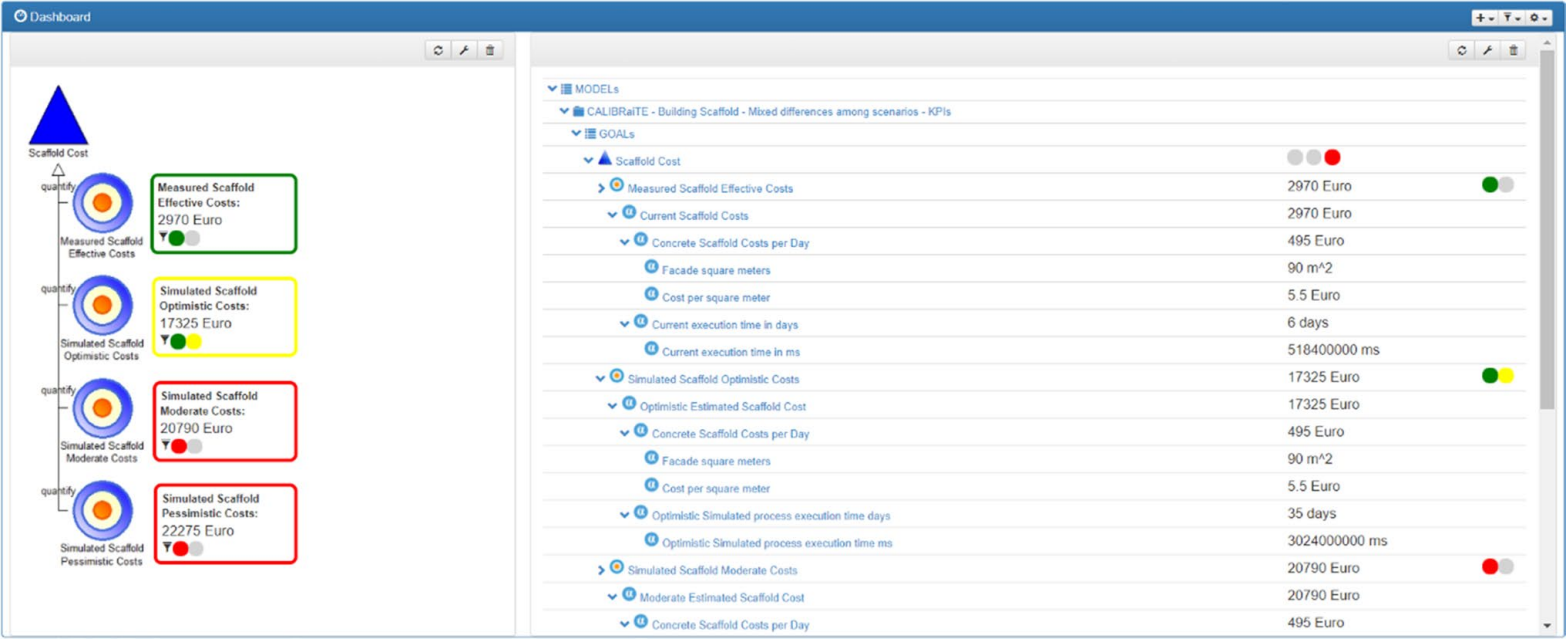
Screenshot of the experimental DSS dashboard prototype (without reliability indicators). *Left side*: overview of the effective costs and the simulated optimistic, moderate and pessimistic costs. Colored margins and circles indicate the extent to which the set threshold has been reached. *Right side*: the detailed KPIs can be accessed through a “drill-down menu”

As part of the cost prediction tasks participants first received an introduction into the renovation project scenario. The scenario consisted of a 1-page description of a renovation project for a 7-story building, which had not been renovated for 30 year. The scenario contained further information about the context and road infrastructure as well as a description of which parts of the building required renovations and to which extent. The participants were then shown the research prototype. Figure 5 shows the experimental DSS dashboard prototype without reliability indicators. The prototype was built on a decision support environment for renovation project managers [29], which maps the Plan-Do-Check-Act framework [37, 68, 110] to typical phases of a construction project. For each of the project phases, one specific dashboard was available, providing decision support through the comparative display of different cost scenarios: effective costs with the currently measured costs and three simulated scenarios differing in the extent of estimated costs (optimistic, moderate and pessimistic). Colored margins and circles in Fig. 5 indicate the extent to which the set threshold has been reached. On the right side, the detailed key performance indicators (KPIs) could be accessed through a “drill-down menu”.

**Fig. 6:**
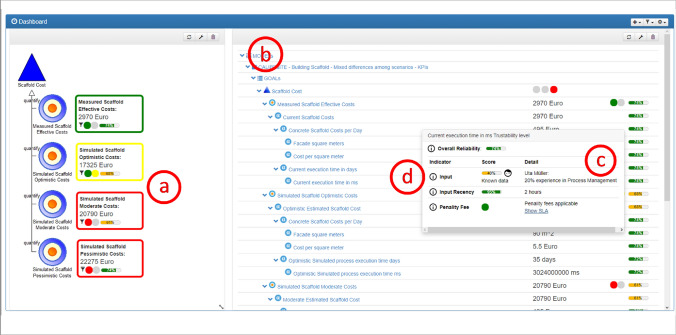
The experimental DSS dashboard prototype with reliability indicators. *Left side*: overview of the effective costs and the simulated optimistic, moderate and pessimistic costs. Colored margins and circles indicate the extent to which the set threshold has been reached. On the *right side*, a drill-down menu offers the detailed inspection of scenarios and KPIs. On the very right side, a reliability indicator is shown for each KPI. When moving the cursor over the indicator, an explanation window shows the underlying information on how the reliability estimation has been derived

The prototype version with reliability indication shown in Fig. 6 is an adaptation of the above prototype by Mirnig et al. [72]. Reliability was presented on different granularity levels: a summary reliability score on the left (a) and a reliability score for each of the KPIs on the right (b). Furthermore, explanations (c) were integrated as a dataset in a popup window (d). More details about the different granularity levels and the content of the explanations is described in Sect. "Strategies for trust calibration".

**Fig. 7:**
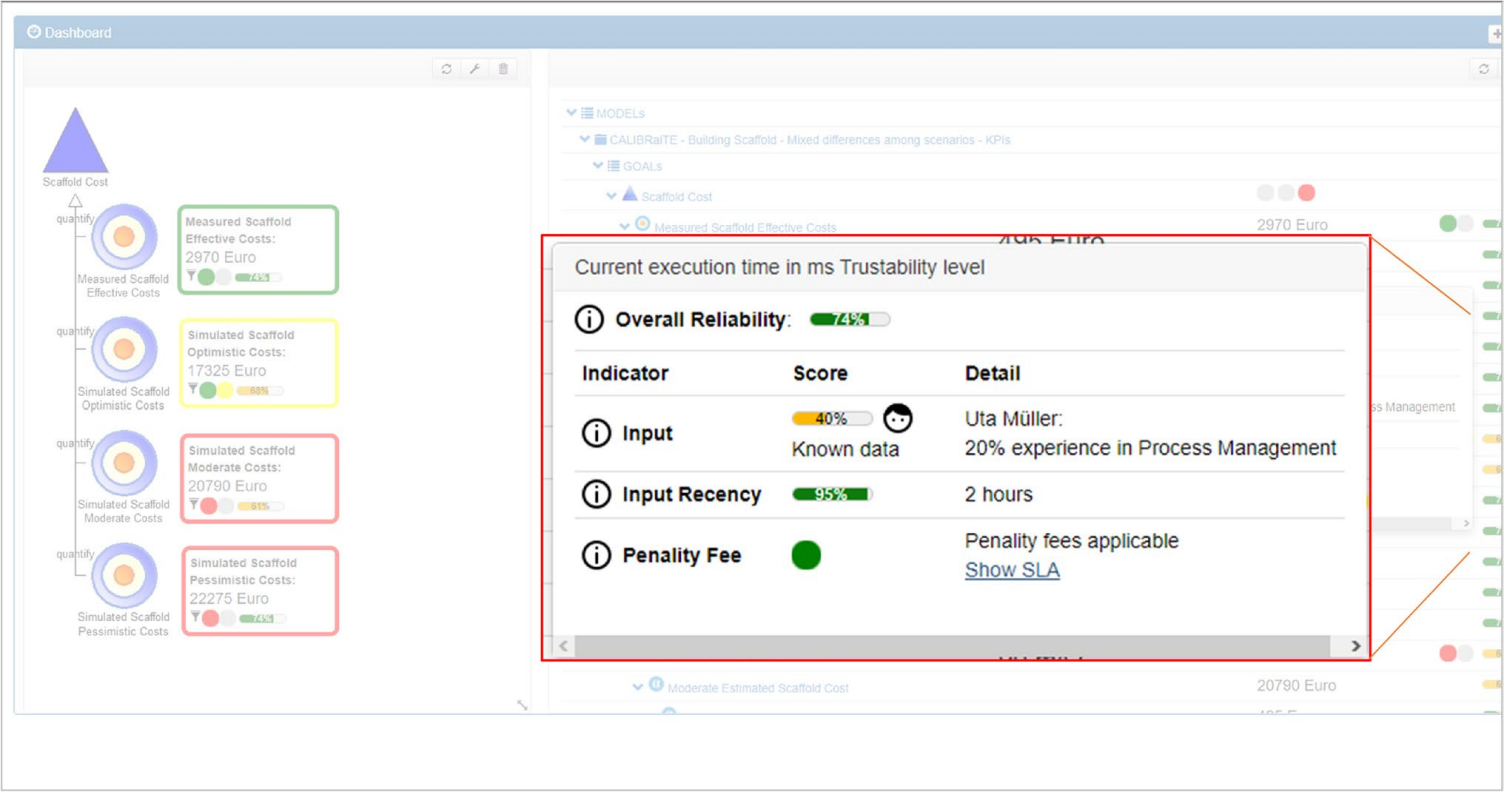
Example for the reliability explanation. The three different information types used in the prototype to model reliability- input, input recency, and penalty fee—are shown in form of a score and text-based explanation details are provided

In order to investigate the effect of reliability indication on decision strategies (RQ1), participants were asked to predict the costs of a renovation project phase and to provide a rating on the confidence in their decision. The cost prediction phase was organized as a 2 × 3 within-subjects experimental design, with the factors ‘confidence indicator’ (present vs absent) and ‘data reliability’ (high, medium, low). Each participant was confronted with each of the resulting six combinations of presence of reliability display and reliability level. The order of these six alternatives was varied systematically across participants.

In the high reliability condition, all KPIs in all scenarios had a high reliability. Vice versa, in the low reliability condition, all KPIs in all scenarios were modeled with a low reliability. The medium reliability condition contained varied reliability levels across the scenarios and across the underlying reliability factors (see Fig. 6 as an example, more details are provided in Sect. "Strategies for trust calibration". The medium scenario was also used as a way to discuss the subjects’ reactions and reasonings on the different forms of providing explanations. The interactions and experiences the participants made in this phase served as the basis for the two subsequent phases where participants reflected on trust and acceptance, as well as on their preference with regard to the future design of reliability indicators.

**Table 3:** Participants’ strategies for predicting the renovation costs in prediction situations with reliability indicators versus situations without reliability indicators

	DSS without reliability indicator	DSS showing reliability indicator
1	Moderate scenario	Moderate scenario
3	Could not make a prediction	Scenario choice oriented to reliability indicators
*4*	Could not make a prediction	Could not make a prediction
5	Moderate scenario	Scenario choice oriented to reliability indicators
6	Moderate scenario	Moderate scenario
7	Could not make a prediction	Scenario choice oriented to reliability indicators
8	Could not make a prediction	Moderate scenario
9	Moderate scenario	Moderate scenario
10	Could not make a prediction	Could not make a prediction

### Inquiry on trust and acceptance

The effect of reliability indicators on trust and acceptance was first assessed quantitatively via two 5-point Likert-scale items. These items were based on the “Trust in Automation” scale by Körber [51] and were adapted to the scope of predictive DSS (in some cases removal and in others rephrasing of scales to fit embodied and/or safety-critical systems) Furthermore, in order to assess acceptance, the three main aspects of the TAM were added to the questionnaire, namely ease of use, usefulness and intention to use. As outlined earlier for each phase we supplemented the quantitative data with qualitative explanations. Here participants were asked to justify and explain their ratings.

### Inquiry on information type and granularity

In this final phase of the study, to address RQ3, participants were asked to share their preferences with regard to granularity and type of information for reliability indication. For each of the three granularity levels per KPI, consisting of a summary score reliability indication and explanations (compare Fig. 6, participants were asked by the interviewer to provide a Likert-scale rating of whether or not they would agree to the statement that they would like to see the respective level of granularity in the user interface. They were also asked to justify their statement. Figure 7 provides an example for the explanations that were made available for users when rolling with their cursor over a specific summary or KPI-related reliability score.

**Fig. 8:**
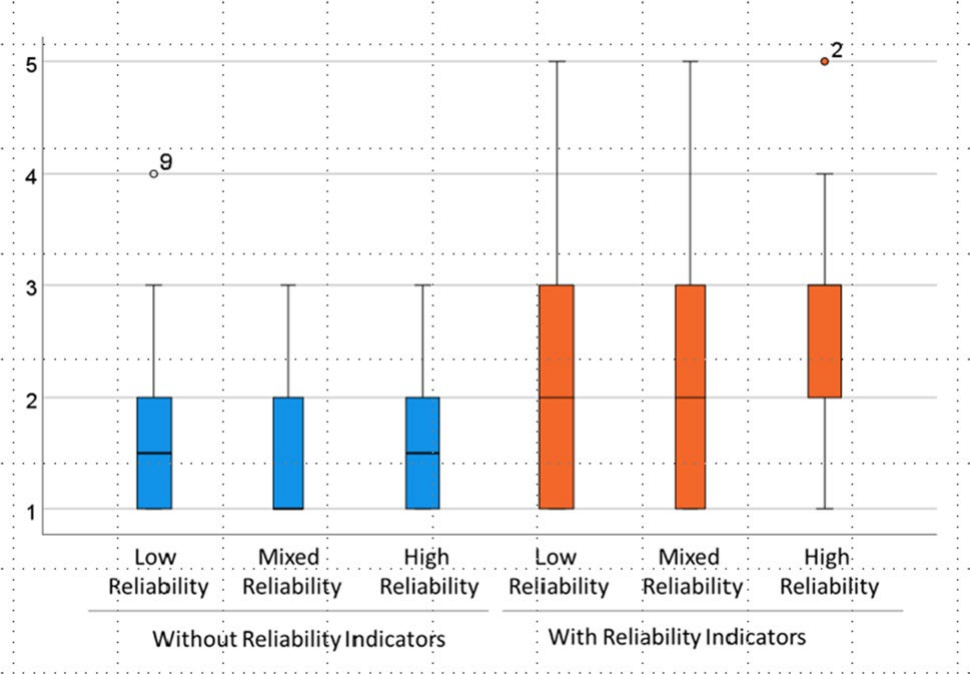
Boxplot diagram (median, interquartile ranges, and outlier cases) for the participants’ confidence in their predictions in the six scenarios under investigation: low reliability, mixed reliability with and without reliability. 1 = very unconfident; 5 = very confident. For the low and high reliability scenarios, data from 10 participants was available; for the mixed reliability scenarios, data from 9 participants was available

In order to capture the preferences on the three chosen input types, participants were asked to provide for each of them a weighting in percent, where the sum should add up to 100% per participant. They were then asked an open question about which features, in addition to the input type, recency, and penalty, they would like to see included in the interface for judging data reliability.

## Results

In this section the findings of the study are presented chronologically per research phase. First, participants approaches, views experiences and expectations in response to the cost prediction tasks are summarized (Sect. “Cost prediction strategies and confidence”. Then, in Sect. “Trust and acceptance”, the results of the inquiry on trust and acceptance are presented, before Sect. “Type of granularity and information” then describes the findings related to the compared information-type and the granularity of reliability indication in the interface.

**Table 4:** Frequencies of ratings for participants’ confidence in their predictions in the six scenarios under investigation: low reliability, mixed reliability with and without reliability. 1 = very unconfident; 5 = very confident. For the low and high reliability scenarios, data from 10 participants was available; for the mixed reliability scenarios, data from 9 participants was available

	Without reliability indicators			With reliability indicators		
	Low reliability	Mixed reliability	High reliability	Low reliability	Mixed reliability	High reliability
5—Very confident	0	0	0	1	1	1
4—.	1	0	0	0	1	1
3—.	1	1	2	2	1	4
2—.	3	3	3	4	3	2
1—Very unconfident	5	5	5	3	3	2

**Table 5:** Participants confidence in their predictions. Summaries of test conditions without reliability indication (middle column) versus conditions with reliability indicators (right column): median, mean, minimum and maximum (1 = very unconfident; 5 = very confident), and comparisons between different reliability levels

	Participants confidence in their predictions	
	Situations without reliability indicators (low, mixed,high reliability)	Situations with reliability indicators (low, mixed, high reliability)
Median	1	2
Mean	1.6	2.4
Minimum	1	1
Maximum	3	5
Difference low-mixed-high (Friedman test)	Chi^2^ = 2.0	Chi^2^ = 4.8
	*p* = 0.37	*p* = 0.09
Difference low-high (Wilcoxon test)	Z = −1.0	Z = −1.9
	*p* = 0.317	*p* = 0.059

### Cost prediction strategies and confidence

Results indicate that displaying a reliability indicator seems to help users to provide a cost estimation: Table 3 provides an overview of participants’ strategies for estimating the renovation costs. In the conditions where data reliability indicators were shown (middle column), four participants consistently chose the moderate cost scenario, and four participants adapted the selection of the pessimistic, moderate and optimistic scenarios to the respective reliability level thus using the indicators for calibrating their reliance on the provided information in the DSS. The other two participants stated that they could not estimate the costs within the given setting. In the decision situations in which the DSS did not provide a reliability indicator (right column), four participants chose the moderate scenario and six could not provide a cost estimation (twice as many as when reliability displays are shown). Four participants opted for the moderate scenarios regardless of whether a reliability indicator was shown or not.

The main reasons for choosing the moderate scenario by the participants seemed to be that they were unsure because they felt they were missing underlying data, especially about the progress of the project, as well as contextual variables such as the current weather. Due to this uncertainty, they therefore made the “Solomonic decision of going for the middle” (P9). One reason from a practical perspective was that “costs beyond the moderate scenario would not be acceptable” (P1). They then stuck to their decision, because they felt that the cost structure was quite similar among the scenarios. The two participants who could not make a decision in either scenario (neither with nor without reliability indicators) stated that they were missing details about the underlying performance and progress, without which they could not match the costs. Three of the four participants who oriented their choice towards the reliability indicators, stated that they could not make a decision in the situations where no reliability indicator was shown.

In general, as per Fig. 8, the reported perceived confidence when supported by a reliability indication was 2 out of 5 (median) and 2.4 out of 5 (mean). This was higher than the one *without* a reliability indication (mean: 1.6; median = 1). A Wilcoxon test comparing the conditions with and without indicators (mean of the three conditions low, mixed and high reliability) resulted in an asymptotic statistical error probability value of *p* = 0.068 (Z = −1.8). A Wilcoxon test comparing between the high reliability situations (the one with reliability indication versus the one without) resulted in a significant difference of *p* = 0.039 (Z = − 2.1).

**Table 6:** Summary of scores for the responses to the trust and acceptance scales adapted from the Trust in Automation questionnaire (TiA, [51]l) and the TAM framework [22]. 1 = do not agree at all; 5 = very much agree. For both types of DSS (with and without reliability indication), mean (M), median (MD), minimum (Min) and maximum (Max) values are presented. Understanding/predictability and propensity to trust were composed of 2 and 3 subscales, respectively. For the comparison between these, in the right part of the table the mean and median differences ($\Delta$M; $\Delta$MD) and the Z- and p-values for the Wilcoxon tests (two-tailed) are shown. Since the scale ‘propensity to trust’ refers to an initial attitude not related to the actual system under investigation, the values for both alternatives are identical, therefore a comparison is not applicable

		Without reliability indication				With reliability indication				Comparison			
		M	MD	Min	Max	M	MD	Min	Max	$\Delta$**M**	$\Delta$**MD**	Z	p
TiA1	Reliability/competence	2.3	2	1	4	3.6	4	1	5	1.3	2	−2.3	.03*
TiA2	Understanding/predictability	1.9	2	1.5	3	3.1	3	1.5	4	1.2	1	1.8	.07
TiA3	Intention of developers	3.5	3	3	5	3.5	3	3	5	0.0	0	0.0	.29
TiA4	Familiarity	3.1	3	1	5	2.6	2	1	5	-0.5	1	1.1	.29
TiA5	Propensity to trust	3.4	3	1	5	3.4	3	1	5	n/a	n/a	n/a	n/a
TiA6	Trust in predictive system	2.2	2	1	4	3.0	3	1	5	0.8	1	1.4	.17
TiA7	Trust in estimation of data quality	2.7	3	1	4	3.4	3	2	5	0.7	0	−1.6	.10
TiA8	Trust in correctness of recommendation	2.3	2	**1**	**4**	**3.2**	**4**	**1**	**5**	**0.9**	**2**	**−1.6**	**.10**
TiA9	Trust in good decision support	2.4	3	1	4	3.4	4	1	5	1.0	1	−1.8	.07
TAM1	Usefulness	2.7	2	**1**	**5**	**3.8**	**4**	**1**	**5**	**1.1**	**2**	**−2.0**	.04*
TAM2	Intention to Use	2.7	3	1	5	3.6	4	1	5	0.9	1	−1.4	.18
TAM3	Ease of Use	2.9	3	**1**	**4**	**3.1**	**3**	**2**	**4**	**0.2**	**0**	**−1.0**	**.32**

Apart from citing the unusual situation of being confronted with predicting the costs of an unfamiliar construction project exclusively based on a dashboard, one important mentioned reason was that the participants were missing some important types of information. Especially the performance-related data about the progress on the construction site, and to be able to match these with the costs, were indicated as missing by several participants. Furthermore, participants would have also appreciated a graphical representation of a timeline and richer spatial data (as known from BIM systems) in order to get a more fine-grained understanding. On the other hand, the prediction scenarios were seen as a valuable support for decision planning (Table 4).

As a next step the effect of reliability indicators on participants tendency to calibrate their trust was investigated. We anticipated that in case of higher trust calibration, increased confidence is measured for the high reliability scenarios and lower confidence is measured for the low reliability scenarios. The results (see Table 5 show that while reliability differences did not statistically differ in the standard conditions *without*reliability indication (*p* > 0.3), in the conditions *with *reliability indication the differences between all three reliability levels is *p* = 0.09. We conclude that the presence of reliability indicators did enable higher confidence in the users’ own predictions as opposed to when no reliability indicators are shown.

### Trust and acceptance

Table 6 summarizes the results of the trust and acceptance questionnaire. Propensity to trust refers specifically in this context to an attitude and accordingly was captured in our study only once and therefore has the same values for both system alternatives. When reviewing the system’s experienced reliability and competence, most participants stated that they could not easily estimate whether the system was reliable, as they have only experienced it for a short time and as they were missing some important information about the actual timeline, the source of the information, and project performance indicators. A general trend was that the alternative with reliability indication was scored as more reliable and capable than without indication. Some participants stated that it is difficult to make a prediction without an indication about the reliability (P2): “If there is no reliability indicator, it is difficult to measure the prediction (P2)”. As the difference between the two alternatives is significant on the 5% level, Z = −2.3, *p* = .03, the effect of the reliability indicator in the context of this system can be regarded as robust.

In respect of the overall familiarity with predictive DSS for construction management, none of the participants knew of a similar dashboard oriented process management system as the one presented. In a wider sense participants mentioned cost simulation programs for offer generation (P1), excel-based data management (P8), generic management dashboards (P9) or risk management systems in other areas apart from cost-related process management (P10) and general predictive analytics software (P5). Regarding the question on whether participants have already experienced reliability indicators in their work context, 7 of the 10 participants stated that they did not yet encounter something similar. P1 reported of some information of whether a certain KPI quality assurance has been performed. P2 mentioned that he has seen much about a color code. The closest that participants could think of were the consultation of pricing indices provided by governments or associations (P3), or the evaluation of the performance description in the contract before the project is conducted (P4). A frequent comment already mentioned in Sect. “Cost prediction strategies and confidence” was that the richness and transparency of data in the background, such as provided through BIM or also other tools, such as Excel, would help to understand where the data comes from and how trustworthy it is.

Several participants mentioned that trust is built over a longer period of time and is also related to the manufacturer, provider and brand. Participants stated that if they had built trust from such previous experiences, they would also trust the system in this situation. For example, P1 stated that he would “not trust the system at once but would also not rule out the possibility of trusting it [in the future].” The maturity of the system also plays a role: “In principle I completely trust the system, if the system is market-ready and out of the beta-product state” (P6). Another important aspect is the general complexity of the construction sector, leading participants to limit their trust in a predictive DSS: “my limited trust is not primarily due to this tool—it is just that you have to live with so many surprises, which does not let you rely on just one system” (P9). Still, the missing information about project progress and performance reported above led to a loss in trust with several participants (especially P4 and P10).

Regarding the difference between the presence and absence of reliability indication, several participants stated that their trust was increased if they have trust cues—or put in another way: “if there is no indicator then I cannot trust it at all” (P2). For those persons who based estimation of reliability exclusively on the “facts” and calculations (especially P4 and P10), the presence or absence of reliability indicators consequently did not make a difference. Overall, the score for trust in good decision support was higher in the presence of reliability indicators than in their absence.

### Type of granularity and information

**Fig. 9:**
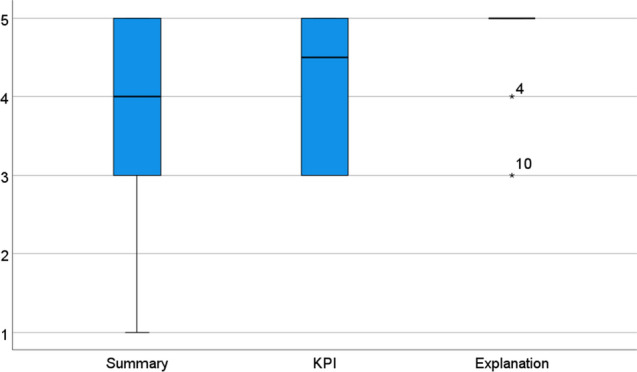
Boxplot diagram (MD, quartile range, outliers) summarizing participants’ agreement to the statement that they would like to see reliability indicators with the respective level of granularity in the interface: as a summary for the whole scenario, for each relevant KPI, and explanation of the underlying reliability factors. 1 = would not at all agree; 5 = would fully agree

Figure 9 summarizes that all three levels of reliability indication granularity were highly attractive for most of the participants, and Table 7 (left side) shows in more detail the scores provided by the participants. As can also be seen, the most convincing form of displaying reliability was the background information (MD = 5). This was consistent with the desire expressed by the participants to get deep insights in the data if they needed to.

**Table 7:** Overview of results per participant on the granularity of reliability indication (left side) and the weighting of the underlying data reliability factors (right side). Granularity of reliability indication: participants’ agreement to the statement that they would like to see reliability indicators with the respective level of granularity in the interface: as a summary for the whole scenario, for each relevant KPI, and explanation of the underlying reliability factors. 1 = would not at all agree; 5 = would fully agree. Data reliability factors: For the three factors input type, input recency and contract penalty, participants were asked to provide percentages adding up to 100% per participant (P4 did not provide percentages)

Job description involvement phases		Preference for granularity of reliability indication			Data reliability factor weighting (%)		
		Total	per KPI	Expla- nation	Input type	Input Recency	Contract Penalty
1	BIM Specialist *initiation, planning, education*	5	5	5	75	20	5
2	BIM Specialist research, education standardization	5	5	5	60	30	10
3	BIM Specialist planning phase	4	5	5	40	30	30
4	Corporate BIM Strategist strategy, project monitoring	1	3	4	nd.	n.d.	n.d.
5	Construction Manager/ Consultant *strategy, project monitoring*	4	5	5	50	25	25
6	Project Manager construction execution	5	3	5	30	50	20
7	Project Manager *construction execution*	5	4	5	70	25	5
8	BIM Manager BIM coordination, management support	4	4	5	70	10	20
9	Contract and Risk Manager offer, planning and contract	1	3	5	50	30	20
10	BIM Manager offer, planning, contract, execution	3	5	3	60	30	10
	Median	4	4.5	5	60	30	20
	Mean	3.7	4.2	4.7	56	28	16

The indication of reliability displays on the KPI level was seen as important (MD = 4.5) in order to be able to compare in detail the reliability. There were different views on the level of detail to be shown. While on the one hand there were doubts that not all details for the KPIs may be needed (P7), on the other hand even a higher granularity was asked for (P4). For the summary score, the median was still high (MD = 4), there was the strongest disagreement and the score was lower than one for the explanation, Z = −2.1, *p* = .04. Some participants said that it is important to get this first overview information: “this should be the result, the most important what should come out; while one should have the opportunity to dig deeper to get a reliable judgment, but at first there should be this summary!” However, there was also skepticism, as the complexity of the situation would often not allow for providing one single value for reliability, or as P4 put it: “this would be the world formula. There are so many criteria; for example, there are more than 50 contract criteria.” A noteworthy requirement that was mentioned to be important for assuming validity of an overall reliability indicator was that the factors and their weighting can be customized by users for each project.

### Factor weightings and additional factors

**Fig. 10:**
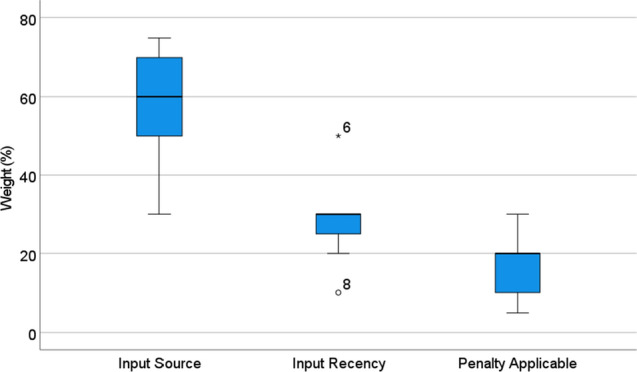
Boxplot diagram showing the weighting percentages (Median, quartile ranges and outliers) that were allocated to the three factors “input source”, “input recency” and “penalty applicable”

As introduced in the previous section the factors used for calculating the overall reliability were input type, input recency, and whether a penalty is applicable for the respective KPIs. Table 7 (right side) shows how participants weighted these three factors by themselves (P4 did not feel prepared to provide a score, due to the complexity and context-dependency of the matter). Eight of the nine participants regarded the input type to be most important, accounting for a median weight of 60% (M = 56%). The input delay was provided with the second-highest weighting by eight participants, receiving a median weight of 30% (M = 28%). The penalty was deemed by 6 participants to have the lowest weight, two participants provided it with the same weigh as input delay, one participant gave it a higher weight than the delay (MD = 20%, M = 16%). The distinct weightings are also illustrated in Fig. 10.

The input source was seen as a central element by the participants for assessing reliability. One important aspect mentioned by most of the participants was that one trusts the input more if it is provided automatically, through a machine. P2 mentions in this regard: “if it is plan data, this data should be transferred from some form of model basis. So, this model basis should be close to as-built, not as-planned.” Reliability explanations that indicated the role and professional experience of the person who entered were seen as important cues for decision for those persons who generally calibrated their decisions towards the reliability indicators (P2, P3, P5, P7).

However, there were also critical voices towards overgeneralizing roles or experience time: “sometimes the trainee is the construction supervisor. The personal experience is not necessarily so highly decisive” (P4). Also, parameters such as the number of years were deemed more important than the years of experience (P7). P10 mentioned that experience in percent is not a reliability cue for him. “If you look at today’s CVs it is difficult to tell what the person really knows.” Instead, the role in the project, however, was very important to him: “it makes a difference if it is an auditor or the service provider him- or herself”, and “whether the person has access to all the data flowing back, that is, whether the person knows about the current performance status on site” (P10).

The complexity of the data also had an important role in deciding whether human or machine input types are preferred: “it depends on the simplicity of the data (e.g. intellectual), then it is up to the human being. In the case of redevelopment, the human input is more credible, because the complexity is higher, and the data situation is more unknown” (P5). P2 noted in this respect: “if have to decide between an AI algorithm and a 100% experienced engineer, I would rely on the human more than on the AI. This project is renovation, in this domain there are many factors that computers cannot measure. If it is just a normal apartment without any extra factors, I would believe a machine more”.

The amount of data also played an important role, and therefore participants welcomed situations where it was indicated how many construction projects the respective recommendation was building upon (P2, P7). While not seen as an absolute necessity, also artificial intelligence processing methods were deemed suitable by the majority of the participants. However, there would need to be a clearer indication as to where the data comes from -the most important distinction being whether it comes from own or external projects.

Regarding input recency, there was a frequent comment that the thresholds should be adjusted from counting the hours towards a longer time span, e.g. working days (e.g., green up to a 2 days delay, yellow for 3-4 days, and anything above this as red). The change rate of the data should be considered here as well: “if the data base does not change much, for example, if it never changed in the last few day, then the reliability should be higher.” (P9). Furthermore, in larger projects, customization of the time thresholds should be possible, which would also enable the project manager to reflect on reliability issues (P10). With respect to the reliability cue about whether a contractual penalty is associated with a certain KPI was least intuitive, but when it had been explained, this appeared worthwhile for many participants: “it does not harm to know which KPIs have an impact on penalties”.

Beyond input type, input recency and contractual penalty, further factors appeared to be important. One related comment was that an indication about previous quality checks and approvals would be worthwhile (P1): “it should be shown whether it is an authorized BIM-model with checked model data, e.g. by dedicated software like Solibri^1^”. In this regard, it would be important to acquire as much as possible real-time information coming from the system which does not have to be entered at all, but generate itself, e.g. the Current Time: if the framework would send such an information itself (IoT). If by means of digital information (3D measurement), certain things are automatically validated, quality management would already come out of another method (e.g., photogrammetric data), without a human being gives the input.

Finally, some of the responses were related more to risk management and decision-relevant information. For example, it was suggested that contextually relevant data, such as weather forecasts, should be available to help to dynamically optimize planning. Specifically on price management, it was recommended to use indices such as national construction cost index, which also factors in the location, inflation and time of year.

## Discussion

The findings of this exploratory study provide first insights on whether and how trust calibration in predictive DSS can be supported in process management tasks generally, and in the complex application context of building renovation in particular. In this section we will discuss the effects of reliability indication that this study identified in the context of previous literature, starting with a focus on users’ prediction strategies and their confidence in the predictions (thus addressing RQ1), followed by a discussion on trust and acceptance related to the overall system usage (RQ2). Finally, the findings related to the compared information type and granularities are discussed and put into the overall research context (RQ3).

### Effects of reliability indication on prediction strategies and prediction confidence (RQ1)

In the decision situation investigated in this study, where construction professionals estimate costs for a renovation project, reliability indication appears to increase the preparedness to make a first statement on future cost developments. 80% of participants were willing to predict costs when they had reliability indicators at their disposal—as opposed to 40% of participants who were not provided with these reliability indicators. Especially users (half of the participants in this study) tended to be more confident in their own decisions when reliability indicators were shown. The study results, and especially the fact that some participants refused to provide an estimation are of a novel quality and cannot be easily compared with prior research that typically features narrow task definitions and well-defined input options for users. The current study conditions included more uncertainty for the participants, as they had to provide a cost estimation without detailed knowledge on all relevant dimensions of the project, especially the progress in terms of performance.

The availability of reliability indicators appeared to motivate a subset of users to calibrate their trust, that is, to adapt their reliance on the system depending on its indicated trustworthiness. In the study, 40% of the participants adjusted their decisions in accordance to what the reliability indicators showed. The effect that people adjust their decisions based on the reliability was observed in other studies as well in other system and usage contexts (e.g., [4, 5, 66, 114]. The strategy chosen by participants to strongly orient towards the different scenarios when predicting cost (pessimistic, moderate or optimistic simulation cost scenarios) and to select one of them, correspond and further extend [74] that presenting alternatives can be supportive in DSS (confer Sect. "Strategies for trust calibration". If such alternatives are provided, it appears that users do not have to calculate everything on their own. A further finding from the analysis of “users’ thinking aloud” comments is that if there are differentiated data reliability values attached to the different scenarios, users can better facilitate the decision-making process, as compared to a system where all reliability values are the same or if there is no data reliability value. Thus, at least for a low number of alternatives investigated in this study appears to outweigh risks of choice overload, which is consistent with the state of knowledge in recommender systems research [10].

### Effects of reliability indication on overall trust and acceptance (RQ2)

As summarized in Sect. "Trust and acceptance", the findings suggest positive effects of reliability indication on several aspects of overall trust and acceptance, at least for situations of first-time usage with imperfect project progress information available. While this finding is the first of its kind for predictive process management DSS of systems, it also corroborates many studies with more narrowly defined tasks from other application fields that also attest clearly positive results of reliability indication (see a collection of related studies in Sect. "Effectiveness of trust calibration communication", 2.5). Both ratings and comments from participants indicate that there are no significant concerns by prospective users against reliability indication. However, users who focus on details of project progress data, cost calculation and risk management, tend to pay less attention to reliability indicators.

The study also provides implications for the further operationalization of trust and acceptance measurement with regard to the comparative evaluation of reliability indicators in predictive DSS. Not all of the items adapted from the ’Trust in Automation’ scale [51] could be transferred from the wider scope of automation systems towards the less dynamic and exposed nature of DSS usage. Thus, although a shorter questionnaire has its merits, further items may need to be developed, for example it is suggested to include a reliably measure as a dimension. The dimension most discriminating between the absence and presence of reliability indicators was ‘Reliability/Competence’, followed by ‘Understanding/Predictability’. The dimension ‘Propensity to Trust’ appeared to be difficult to answer and naturally did not provide a means for comparison. The dimension most difficult to integrate in the study inventory was ‘Intention of Developers’, mainly because the system was still a research prototype presented by a member of the development team. Thus, in case of an experimental research study like the one conducted here, this scale may be omitted.

Familiarity as such was a highly important aspect to be investigated in the study, as this helped to define its (low) level of penetration in current systems and thus, in combination with the high demand by users, underlines an innovation need. However, the role of familiarity within the comparative measurement of effects reliability displays on trust, is quite ambivalent. ‘Familiarity’ was the only dimension where the system without reliability indicator received a higher score, as this feature was less well known. Thus, the little familiarity here rather expresses the novelty of reliability indicators and should be less regarded as a threat for limiting trust. In line with a critical note by Körber [51] on the application of his questionnaire, familiarity could be omitted in a future measurement instrument that measures the effect of reliability displays. Of the four different trust scales, the item ’Trust in good decision support’ was most salient in the usage context of predictive DSS in renovation process management. From the items included that mention overall trust, it appears that due to its salience this scale may actually be most suitable to measure the tasks performed in this study.

The results related to technology acceptance factors indicate that reliability indicators significantly increase the perceived usefulness of a process management system. This reflects well the above discussed results on the effects on prediction strategies and confidence: A predictive DSS is seen as more useful if it provides reliability indicators that support users in ’daring a prediction’ as this helps to adjust the prediction and confidence of the underlying data quality. However, reliability indicators do not increase the ease of use, as this characteristically is more influenced by user interface design. Reliability indication also does not appear to be a robust antecedent for the intention to use a predictive DSS.

Previous comparative studies on the added value or reliability indicators have not included these major technology acceptance factors in their analysis, thus this study provides a novel contribution in this regard. The absence of an effect on the intention to use highlights the complexity and multifaceted nature of acceptance and trust: Participants’ comments show that apart from reliability displays, many more aspects play into the formation of trust. Here, especially the performance in previous situations plays a decisive role. Since participants were exposed as part of this study to a new tool and fictitious usage scenarios, participants could not rely on these pre-assumptions. The long-term formation of trust and intention for future use is a well-known factor discussed in the literature (e.g., Lee and See, 2004).

### Preferable type and granularity of reliability information (RQ3)

A novel finding of this study is that predictive DSS for process management should provide reliability indication on several levels, aspects and granularities of the user interface. This study demonstrated that it is, for example, not sufficient to attach a reliability indicator only to the predicted overall project costs. Potential concerns about cluttering the display with additional information [80] are not supported by the study findings: ease of use was not affected by the reliability indications and positive comments about reliability indicators by far outweighed the critical ones. Participants’ responses point towards other aspects that may be more decisive for a high ease of use, such as further visual features for data exploration and a consistent integration with the process management dashboard in a wider project monitoring software environment.

As for the precise reliability information to be included in the user interface, the strongest preference was found for reliability explanations which provide background information on the factors contributing to overall reliability scores. While this identified preference is in line with the growing body of research arguing for providing explanations for decisions of DSS (including the ’explainable AI’ research stream, see [79], the approach in this study is novel, as it investigates different forms of communicating the factors underlying the derivation of a reliability estimation.

Furthermore, factors for data reliability determination were investigated in a systematic empirical study for the first time. Based on this research more specific requirement for the design of reliability explanations were derived. More specifically, the relevance of the requirements for communicating data reliability gathered and prioritized by Mirnig et al. [70] was confirmed, i.e., user expertise-based user roles, input recency, and contractual penalty. The soundness of representing data reliability requirements has been presented beyond the purely conceptual level, as their integrated presentation in the user interface has been demonstrated and evaluated in the user study. The study confirms that also several further information types suggested by Mirnig et al. [70] as relevant factors for determining reliability are desirable by users to inspect data requirements. This especially concerns an indication on whether data quality checks had been performed beforehand, e.g., by dedicated BIM model checking tools or stage gate process approvals in the construction process.

The requirement in [70] to offer explanations for the underlying information processes was also confirmed, but this was not restricted to AI: on the one hand, this included the wish for even more details on the already presented reliability explanations (e.g., details about the relevance and role of the person providing the input or more cost calculation terminology from the construction domain). On the other hand, explanations for the overall project progress beyond pure reliability estimation are of high necessity. This is in line with the findings by van der Waa et al. [104], who also observed that experts want to have full access over the data to form their own judgment on the DSS’ advice.

## Conclusions

This study closed a gap in current DSS literature by providing explicit reliability indication. The findings gathered in this study allow for a number of conclusions that provide on the one hand suggestions for future application-oriented research and on the other hand forms a basis for an overall set of recommendations. The overarching implication from this study is that reliability indication may not be the most decisive factor for the overall intention to use a DSS system as such, but it clearly adds to its usefulness if properly integrated. In particular, this implication holds for situations in which professionals are first confronted with a project without complete overview of the detailed progress and conditions. In these situations, reliability indication may enable for higher preparedness to provide preliminary estimations and a better calibration to the underlying data quality.

The implications for corporations currently driving their digital transformation by introducing advanced decision aids and BIM technologies is that reliability should be implemented as a key information layer that should be attached closely to all forms of data and that should be readily accessible for a manager being introduced into a construction project. Systems with such a “reliability-indication-by-design” approach are currently neither offered in the construction domain nor in most other application fields of process management systems. Introducing “reliability-indication-by-design” represent an innovation with a competitive advantage both for the construction companies applying them and for the software companies providing them.

More specifically, the presented work provides novel prescriptive insights into how to design reliability indication in process management systems. In line with the overall implication of persistently offering reliability indication in a DSS, especially if complementary rather than repetitive, relevant information should be provided at several different abstraction or granularity levels of the user interface. The exploration and inquiry of information granularity implies that exclusively providing one single reliability indicator at the top level (e.g., attached to the predicted overall project costs) may not support trust formation in most professionals. Besides the practitioners’ realism about the feasibility of finding the “world formula” for calculating an overall reliability score, pure reliance on summary scores on dashboards does not correspond to their usual working style of literally “drilling deep” into the data, in order to check for plausibility, potential risks, credibility of service providers, and other aspects.

Presenting several reliability indicators in the user interface at a lower abstraction level, in association with each relevant KPI, appears to be more helpful than one overall score. However, effectiveness appears to be dependent on the way how this KPI-related reliability indication is offered. Based on the findings, it especially appears important to determine the “relevant” KPIs for each project (or at least project type), in order to reduce repetitiveness and clutter in the interface and thereby support usage efficiency. Such purposeful relevance determination could go as far as only providing reliability indicators for those KPIs, for which an inspection matters most. For instance, in line with the “Pareto Principle” common in the construction industry [6], with an improved user interface users could be inspired to focus on the inspection of those 20% percent of KPIs that account for 80% of a project’s costs.

A key finding of the study is that transparent explanation of a reliability score necessitates an explainable approach for the modeling of reliability. The approach used in this study—to derive and to prioritize a set of factors from interviews with targeted domain expert users that results in three factors: input type, input latency and contract penalty—appears to be promising. For the fundamentally differing input types the target users tend to be interested in seeing more detail than what has been provided in this pilot model, for example, more context-specific explanations: the role, involvement and responsibilities of the person in the respective project are deemed to be more relevant than a summary score of experience in a certain subject matter.

Having stressed the relevance of reliability indication in an interface should not distract from the responsibility of project managers to check for additional risks, plausibility, trustworthiness of contractors and other aspects that are relevant for assuring the success of their projects. Thus, reliability indicators can only provide an added benefit if the DSS are integrated and also have strong capabilities for controlling project progress and quality. According to the views of the involved participants, especially a good resolution and fidelity with regard to the timeline and the spatial representation is important, with both being supported by advanced BIM systems. Participants also found that the reliability explanations investigated in the study can form a useful bridge towards exploration of further underlying data sources and thus support users to drill deep into the data.

### Limitations

While the expert-oriented evaluation study and its mixed-methods approach enabled to gain an in-depth understanding of effects and design requirements of reliability indicators in a rich contextual setting, it should be seen as a first preliminary step. The sample recruiting strategy of systematically addressing representatives of the most relevant actors within the national ecosystem supported the balancing of the sample, but it restricted the geographic spread. Even though all participants had experience with international projects and customers, they may have been biased by the technologies used in the Austrian construction domain and not accounting for the diversity of work cultures [100].

Given the good fit with the current target group profile, the sample size of 10 participants appears satisfactory for the qualitative analysis and a number of very robust effects could be identified, however, with a larger sample more subtle quantitative effects could have been confirmed. Similarly, the evaluation focused only on a single view at a certain step within a larger project, resulting in information being missing that would have likely been available to the project manager of a real project. Whether the missing pieces of information had an impact on the overall confidence estimations of the participants we cannot say with the data available, although we did ensure to not carry forward uncertainties caused by the setup as uncertainties inherent to the process in the analysis.

The methodological paradigm of creating experience prototypes followed in this study is key to HCI research and user-centered research [84]. It provides the opportunity to investigate system concepts early and efficiently, without the need for full implementation. However, for each study, an inherent trade-off is to be performed between the prototype fidelity (i.e., the aspired level of external validity) and the feasibility (in terms of effort and capabilities) to reach this fidelity.

### Future research

The research presented in this paper suggests that communicating reliability can provide added value for process management DSS in complex application domains such as renovation and construction. The study deliberately covered situations of first confrontation with a project, where a main source of information (costs) is available, but other important context is not provided (e.g. performance of the project). Future research should investigate usage situations that encompass longer usage times and that support final decisions based on more complete availability of context information, using, for example, an extended experience prototype that provides integrated simulated data about project performance, spatial and temporal simulations of a fictive project and more risk management procedures. A further extension of this approach would be to allow user customisation of reliability indication in the UI and to investigate the value thereof in order to derive sensible customisation options for the user.

Additional future perspectives include longitudinal investigations to ascertain formation and calibration of trust in the long term, cross-relations to performance, as well as potential changes in UI/output requirements after sustained use of a system with reliability indication. Furthermore, in order to develop a more general concept of reliability indication in intelligent systems, future studies need to look beyond the process management of renovation projects. A first step here is an extension into related “neighboring” application areas such as spatial planning of urban spaces or of nationwide infrastructure. In the end, the design of DSS is strongly context dependent, so valid abstractions on the general level will rely and depend on successful applications in a multitude of different domains.

## Footnotes

1 www.solibri.com.
